# Analysis of helkimo and craniomandibular indexes for temporomandibular disorder diagnosis on rheumatoid arthritis patients

**DOI:** 10.1016/S1808-8694(15)31117-4

**Published:** 2015-10-20

**Authors:** Suzana C. da Cunha, Ricardo Viana Bessa Nogueira, Ângela Pinto Duarte, Belmiro Cavalcanti do Egito Vasconcelos, Renata de Albuquerque Cavalcanti Almeida

**Affiliations:** 1 Dentist - Dentistry School of Pernambuco - FOP/UPE; 2 Specialist and Master in Buccomaxillofacial Surgery and Traumatology - Dentistry School of Pernambuco - FOP/UPE; PhD Student in Buccomaxillofacial Surgery and Traumatology - Dentistry School of Pernambuco - FOP/UPE; 3 Full Professor of Rheumatology - Federal University of Pernambuco (UFPE); 4 PhD. Postgraduate course Coordinator - UPE; 5 Resident in Buccomaxillofacial Surgery and Traumatology - Hospital Universitário Oswaldo Cruz-HUOC/UPE, Dentist - Dentistry School of Pernambuco - FOP/UPE

**Keywords:** arthritis, rheumatoid arthritis, diagnosis, temporomandibular disorder

## Abstract

**Summary:**

Aim: The aim of this study was to evaluate the use of two indexes (Helkimo and Craniomandibular) for the diagnosis of temporomandibular disorder (TMD) in patients with Rheumatoid Arthritis (RA).

**Patients and methods:**

The sample was composed of 80 patients divided into two groups: patients with RA and patients without RA. In both groups the two indexes were used. For TMD diagnosis, the following signs and symptoms were evaluated: TMJ pain, limited mouth opening and joint sounds.

**Results:**

Results showed that of the RA patients, 87.1% were females and 12.9% were males. Among the patients without RA, 70% were females and 30% were males. The age of these RA patients ranged between 24 and 78 years. Among patients without RA, the age of the patients ranged between 22 and 72 years. It was observed that the prevalence of TMD was higher in the group with RA (98.6% - Helkimo and 87.1% - Craniomandibular) when compared to the group without the disorder (80% - Helkimo and 50% - Craniomandibular).

**Conclusion:**

In summary, one could conclude that both indexes are capable to diagnose temporomandibular disorders in RA patients, although the Helkimo index is less accurate.

## INTRODUCTION AND LITERATURE REVIEW

It is estimated that at least from 50% to 60% of the population present some kind of TMJ disorder, that is myofacial dysfunction, internal derangement or degenerative joint disease, and females are more affected, especially those between 20 and 30 years of age^1^,^2^, and such fact has deep impacts in otorhinolaryngology because TMJ disorders are often mistaken by earaches.

Rheumatoid arthritis (RA) is the main degenerative joint disorder and is considered an autoimmune disorder of unknown etiology. It is manifested as peripheral and symmetrical polyarthritis that causes joint deformity and destruction because of bone and cartilage erosion. This may happen to major and small joints in association, and happens together with systemic manifestations. Its global prevalence is close to 1%, and women are three to four times more affected than men, with incidence peak between 35 and 50 years^3^, ^4^, ^5^, ^6^, ^7^.

The American Rheumatism Association published, in 1987, a guide for RA diagnosis, according to which the patient must present three or four of the following symptoms for more than 6 months: 1- morning stiffness for more than one hour; 2- arthritis in three or more joints; 3- arthritis in the hands; 4- symmetrical arthritis; 5- rheumatoid nodes; 6- rheumatoid factor present; 7- radiographic alterations^6^,^7^.

The likelihood of RA patients developing TMJ symptoms is related to the severity and duration of the systemic disease^8^, ^9^, ^10^, ^11^, ^12^, ^13^. This fact has been reported since the very term Rheumatoid Arthritis was introduced in 1859, by Garrod, who believed RA had a peculiar tendency to affect such joint^14^. TMJ involvement rate varies between 4.7% and 84%^8^, ^9^, ^10^, ^11^, ^12^, ^13^. According to the literature, the most frequently reported symptoms are: joint pain, edema, joint crepitation, stiffness when opening the mouth and movement limitation^8^, ^9^, ^10^, ^11^, ^12^, ^13^, ^14^.

Many papers point towards the need to have a standardized classification for TMJ disorder's signs and symptoms, and the use of indices is an excellent means to allow disease severity to be individually classified in order to examine the incidence of such problem in a specific population, measure the effectiveness of the therapies employed and study etiologic factors^15^,^16^.

Helkimo was a pioneer in developing indexes to measure the severity of TMJ disorders, as well as pain in this system. In an epidemiological study, he developed an index that was further broken down into anamnesis, clinical and occlusal dysfunction. Through this index, he tried to assess, individually and in the general population, the very prevalence and severity of TMJ disorders in mandibular pain and occlusal instability.^17^.

The study developed by Fricton and Schiffman^18^ aimed at developing a craniomandibular index and test it as to its reliability. Later studies showed that the use of these indexes allowed for a safe evaluation of the signs and symptoms of temporomandibular disorders in the patients investigated^18^,^19^.

The present investigation aims at evaluating the use of two indices (Helkimo and craniomandibular) used for the diagnosis of temporomandibular disorders in patients with Rheumatoid Arthritis, as well as check if there is any significant difference in relation to any of the components from the Helkimo and the Craniomandibular indices.

## MATERIALS AND METHODS

This investigation was carried out in the city of Recife, in the State of Pernambuco, and the studied population was taken from the spontaneous or referred patients to the Discipline of Rheumatology of the Federal University of Pernambuco and the Dentistry School of Pernambuco; and it was approved by the proper Ethics Committee. The sample was made up of 80 patients: 70 coming from the Department of Rheumatology of the Federal University of Pernambuco, diagnosed with RA - according to the criteria established by the American Association of Rheumatology (1987); and the other patients did not have Rheumatoid Arthritis and came from the School of Dentistry of Pernambuco.

In this study we included the patients with RA that came for clinical evaluation and were affected or not by temporomandibular disorders. The patient was considered a bearer of some temporomandibular disorder when he/she presented at least one of the following items: TMJ pain; mouth opening limitation; intra-articular noise. The patients without rheumatoid arthritis included in this investigation were RA-free patients, confirmed by joint exams in agreement with the American Association of Rheumatology (1987). These patients could or could not have temporomandibular disorders.

For data collection purposes, we created a structured form where we wrote down patient identification data and the results from the Helkimo and craniomandibular indices. The form was filled out after the patient had been properly informed on the content of our research and signed an Informed Consent Form.

In the utilization of the Helkimo index, patients were analyzed based on the evaluation of three sub-indices: the first of them is the anamnesis index, based on the different symptoms of dysfunction in the masticatory system (subjective symptoms) reported by the individuals during history taking. Such index may have three different levels:

Ai-0: Made up of individuals free from dysfunction symptoms;

Ai-I: Made up of individuals with mild dysfunction symptoms;

Ai-II: Made up of individuals with severe dysfunction symptoms.

The second is the clinical dysfunction index, which considers a functional evaluation of the masticatory system. In accordance with the presence and/or severity of these clinical symptoms, individuals are assigned a score of 0, 1, or 5 points. The following items were observed: a- Range of mandibular motion; b- TMJ function impairment; c-Muscle tenderness during palpation; 4- TMJ pain during palpation; e- Pain during mandibular movement - only recorded when clearly identified. According to the score attained, the individuals were classified in four groups:

Di-0: 0 points - Individuals clinically free from dysfunction symptoms;

Di-I: 1 to 4 points - Individuals with mild dysfunction symptoms;

Di-II: 5 to 9 points - Individuals with moderate dysfunction symptoms;

Di-III: 10 to 25 points - Individuals with severe dysfunction symptoms.

The third index is the so called occlusal, obtained from an analysis of the individual's dental occlusion. According to the data obtained from each item, the individuals were assigned a score of 0, 1, or 5 points, as follows: number of teeth, number of teeth in occlusion, occlusal interference between centric relation and centric occlusion and joint interference. According to the score obtained, the individuals were classified in three groups:

Oi-0= 0 points - no occlusal or articular disorder;

Oi-I= 1 to 4 points - moderate intensity occlusal or articular disorder;

Oi-II= 5 to 20 points - severe intensity occlusal or articular disorder.

It was necessary to adjust the Helkimo's index because it is not very clear in relation to its anamnesis and occlusal components, and it was up to the researcher to interpret it. As to the anamnesis index, considering the points to be analyzed, the following options were available: no symptoms, mild, moderate or severe symptoms. A patient was considered to be Ai0 (dysfunction symptoms-free) when he/she answered NO to all the questions; AiI (mild dysfunction symptoms), patients who answered MILD or MODERATE; and AiII (severe dysfunction symptoms), those patients that answered SEVERE for at least one of the questions. As to the occlusal index, the value to be assigned for number of teeth, number of teeth in occlusion, occlusal interference in centric relation and centric occlusion; and it is worth mentioning that the value to be used for each one of the situations must be divided by two, since we have two dental arches (upper and lower). A convention was decided upon for assigning zero in the following situations: all teeth present; occlusion with natural teeth; no occlusal interference in centric relation or occlusion; or lack or articular interference; joint interference of the click type. 2.5 points were assigned for each dental arch in cases of total edentulous patients; non users of prosthesis; many points of occlusal interference; crepitation-type joint interference. As to the dysfunction component, it was equally followed, without doubts.

For the craniomandibular index (CMI), subjective and objective exams are made up of the following routines: a) patient identification; b) extra-oral (EO), intra-oral (IO) and neck (NM) muscle palpation; c) observation of the signs and symptoms related to mandibular movements (MM); d) TMJ noise auscultation; e) TMJ region palpation, aiming at detecting tenderness in these areas.

For result analysis, we established a scoring system, represented by the so called “Craniomandibular Index” (CMI), which used procedures in each patient to quantify craniomandibular signs and symptoms, thus allowing for an objective assessment, and particularly, an analysis of the severity of the Myofacial Dysfunction (MD) and the painful dysfunction of the temporomandibular joint (TMJD), for each patient evaluated. In order to analyze symptoms and visual signs during mandibular motion (MM), we observed the patient's general appearance and mandible movement in all motion types. Their limitations and painful aspects were assessed and quantified numerically. For each positive answer from the patient a value (1) was assigned; and for each negative answer, a value (0) was assigned. The set of procedures that reflected changes in muscle sensibility characterizes the palpation index (PI), while the procedures corresponding to mandible functioning, joint noise and palpation tenderness in the TMJ area make up the Dysfunction Index (DI).

In order to calculate the PI, we add up all the positive answers obtained through the palpation of the intra and extra oral muscles and neck muscles, and divide it by the number of events (36). In order to calculate DI, we add up all the positive answers in regards of the mandibular movements, joint noise and sensibility related to joint capsule, and divides it by the number of events (30). In order to calculate CMI, we add up the dysfunction and palpation indices and divide it by two.

For data descriptive analysis, we obtained absolute and percent distributions for the nominal or categorical variables and the statistic measures (minimum value, maximum value, mean value, Standard deviation and the variation coefficient for numerical variables). For the inferential analysis we used the Person chi-squared tests for proportions' equality (or the Fisher Exact Test, when there were no conditions for the Chi-squared test); the Student t test with equal or unequal variances and the F test ANOVA, we also used Tukey paired comparison tests. It must be stressed that we established the variants equality hypothesis by means of the F test for this specific end. The maximum error margin (level of significance) used in the statistical tests decision was of 5%. The data were entered in an Excel spreadsheet, and the statistics software used was the SAS (Statistical Analysis System), version 8.0 for microcomputers.

This study was approved by the Ethics Committee of the University of Pernambuco, under protocol # UPE-033/04.

## RESULTS

Of the 70 patients with Rheumatoid Arthritis (RA), 87.1% were females and 12.9% were males. Among the patients without RA, 70.0% were females and 30.0% were males. The age of RA patients varied between 24 and 78 years, with mean age of 46.05 years, standard deviation of 12.78 years. Among the patients without RA, age varied between 22 and 72 years, mean age was of 35.60 years, standard deviation was 16.51 years. On Table 1, we see patients' distribution according to age range.

**Table 1:** Distribution of patients according to age range (in years)

Group						
Age (in years)	With Rheumatoid Arthritis		Without Rheumatoid Arthritis		Whole Group	
22 a 40	28	40,0	7	70,0	35	43,8
41 a 60	31	44,3	2	20,0	33	41,2
61 a 78	11	15,7	1	10,0	12	15,0
TOTAL	70	100,0	100,0	100,0	100,0	100,0

Table 2 depicts the results from the Helkimo Index components according to group. In this table it is possible to see that the groups present conclusions that are very different in terms of index's components. In analyzing the anamnesis component, we notice that most of the patients studied (53.7%) were classified as AiII. Among the groups, we highlight that the patients classified in categories Ai0 and AiI were correspondently higher in the group without RA when compared to patients with RA (20.0% versus 4.3% in Ai0 and 80.0% versus 50.0% in AiI); and the opposite happened with AiII, which had 45.7% of the patients from the group of patients with RA, without positive results in disease-free patients.

**Table 2:** Evaluation of Helkimo's Index components according to group.

Group							
Component	With Rheumatoid Arthritis		Without Rheumatoid Arthritis		Whole Group		p value
	n	%	n	%	n	%	
Anamnesis component							
Ai 0	3	4,3	2	20,0	5	6,3	p(1) = 0,0038*
Ai I	35	50,0	8	80,0	43	53,7	
Ai II	32	45,7	-	-	32	40,0	
TOTAL	70	100,0	10	100,0	80	100,0	
• Dysfunction component							
Di 0	29	41,4	10	100,0	39	48,7	p(1) = 0,0073*
Di I	29	41,4	-	-	29	36,3	
Di II	8	11,4	-	-	8	10,0	
Di III	4	5,7	-	-	4	5,0	
TOTAL	70	100,0	10	100,0	80	100,0	
• Occlusal component							
Oi 0	1	1,4	8	80,0	9	11,3	p(1) < 0,0001*
Oi I	32	45,7	2	20,0	34	42,5	
Oi II	37	52,9	-	-	37	46,2	
TOTAL	70	100,0	10	100,0	80	100,0	

(1) - By means of the Fisher's Exact test.

(2) - by means of the Person's chi-squared test.

(*) Significant difference at the 5.0% level.

Still on Table 2, we can see that in relation to the Helkimo dysfunction component, all the normal patients were classified in Di0, while in the group of patients with RA, less than half (41.4%) were classified as Di0; 41.4% in DiI, 11.4% as DiII; and 5.7% as DiIII. For the Occlusal component of this same index, we see that only one RA patient was classified as Oi0, while in the group of patients without RA (normal), 8 were classified in the aforementioned condition. On the other hand, none of the so called normal patients was classified as OiII, while in the group with RA such percentage corresponded to 52.9% of this group's sample. By means of the statistical test, we see a statistically significant difference between the two groups in relation to each one of the Helkimo Index's components (p < 0.05).

On Table 3 we see the CMI standard deviation and mean value according to gender. In this table we see no great differences between both genders for CMI or its components, since the largest difference was recorded for the palpation component, with a value of 0.03; we did not see significant differences among male and female patients in relation to the mean value of this index.

**Table 3:** Craniomandibular, palpation and dysfunction components per gender and total group Mean and Standard Deviation for RA patients.

Gender					
Variables	Statistics	Males	Females	Whole group	p(1) value
• Craniomandibular index	Mean	0,11	0,12	0,12	p = 0,7883
	Standard deviation	0,07	0,13	0,13	
• palpation	Mean	0,04	0,07	0,07	p = 0,6392
	Standard deviation	0,10	0,16	0,15	
• dysfunction	Mean	0,18	0,18	0,18	p = 0,9893
	Standard deviation	0,11	0,16	0,15	

Table 4 depicts mean CMI values, and we notice that they were higher in the age range between 41 and 60 years, and the highest difference was equal to 0.06 for the dysfunction component (0.15 in the age range between 24 and 40 years; and 0.21 in the age range between 41 and 60 years), nonetheless, we do not see significant differences among the age ranges for any of the three variables (p > 0.05).

**Table 4:** Craniomandibular, palpation and dysfunction components per age range Mean and Standard Deviation for RA patients.

Age range (in years)					
Variables	Statistics	24 a 40	41 a 60	61 a 78	p(1) value
• Craniomandibular index	Mean	0,11	0,14	0,11	p = 0,5226
	Standard deviation	0,10	0,16	0,06	
• palpation	Mean	0,06	0,07	0,05	p = 0,8979
	Standard deviation	0,11	0,19	0,11	
• dysfunction	Mean	0,15	0,21	0,16	p = 0,3202
	Standard deviation	0,14	0,17	0,11	

(1) - By means of the F (ANOVA) test.

On Table 5 we see that TMJD prevalence was higher in the group with Rheumatoid Arthritis when compared to the group without RA (98.6% versus 80.0%) and the statistical test indicates a significant difference between the two groups regarding TMJD by the Helkimo's index.

**Table 5:** TMJD occurrence evaluation by the Helkimo's index according to group.

Group							
TMJD by the Helkimo's index	With rheumatoid arthritis		Without rheumatoid arthritis		Whole total		p value
	n	%	N	%	n	%	
Yes	69	98,6	8	80,0	77	96,3	p(1) = 0,0398*
No	1	1,4	2	20,0	3	3,8	
TOTAL	70	100,0	10	100,0	80	100,0	

(1) - by means of the Fisher's Exact test.

(*) Significant difference at the 5.0% level.

On Table 6 we see that TMJD prevalence was higher in the RA group when compared to the group without the disease 87.1% versus 50.0%) and statistical testing points towards a significant difference between the two groups in regards of TMJD by the Craniomandibular index.

**Table 6:** TMJD occurrence evaluation by the craniomandibular index, according to group.

Group							
TMJD by the craniomandibular	With rheumatoid arthritis		Without rheumatoid arthritis		Whole group		p value
	n	%	N	%	n	%	
Yes	61	87,1	5	50,0	66	82,5	p(1) = 0,0123*
No	9	12,9	5	50,0	14	17,5	
TOTAL	70	100,0	10	100,0	80	100,0	

(1) - By means of the Fisher's Exact test.

(*) Significant difference at the 5.0% level.

## DISCUSSION

Rheumatoid Arthritis (RA) is a chronic and inflammatory disease, primarily characterized by persistent synovitis, usually in peripheral joints and symmetrical. Women are three to four times more affected than men^3^, ^4^, ^5^, ^6^, ^7^. The present investigation also found more women affected 87.1% versus 12.9%.

According to the literature3-7 RA prevalence peak is between 35 and 50 years of age, and the age of onset varies between 25 and 55 years, in agreement with this study, in which patients' ages varied between 24 and 78 years, with mean age of 46.05 years. In the present investigation, most of the patients were between 22 and 40 years (35 patients); and 41 to 60 years (33 patients), while only 12 were between 61 and 78 years.

We found it very difficult to observe TMJD prevalence and severity both individually and in the general population by means of the anamnesis, dysfunctional and occlusal components of the Helkimo's index, because of the numerous failures existing. It is not clear how one should calculate the anamnesis and occlusal components of the index, and the researcher is the one who should do it as he/she sees fit, adapting index in question so that it becomes feasible to be used. It also does not allow the attainment of a numeric value for the index; thus, its interpretation bears a subjective character.

Since we did not find in the literature any study with similar methodology, we tried to use these indexes in a sample of patients free from Rheumatoid Arthritis, as a means of comparison. As to the anamnesis component in the Helkimo's index for RA patients, 50% were classified as Ai-I (individuals with mild dysfunction symptoms); 45.7% in Ai-II (severe dysfunction symptoms); while 4.3% were classified as Ai-0 (individuals free from dysfunction symptoms). In the group of patients free from Rheumatoid Arthritis, 20% were classified as Ai-0; and 80% as Ai-I. There was no patient with severe dysfunction symptoms, relating a higher temporomandibular joint involvement in patients with Rheumatoid Arthritis, from a patient's stand point.

As to the dysfunction manifestation among patients with Rheumatoid Arthritis, most of the patients were classified as free from dysfunction or with mild symptoms, and the rest presented moderate to severe dysfunction symptoms. On the other hand, all patients without RA had not dysfunction symptoms, and such fact shows the greater joint and muscular dysfunction severity in patients with Rheumatoid Arthritis, and the most frequent signs are tenderness to palpation of joint and/or muscles and the reduction in muscle movement and joint function. The results found hereby are in agreement with results reported in the literature, showing that there is no relation between patients with and without RA with greater or lesser mandibular opening^8^,^9^,^13^,^14^,^20^.

As far as the occlusal component is concerned, most patients had moderate joint or occlusal disorder (45.7%) and with severe occlusal or joint disorder (52.9%), while only 1.45% did not present joint or occlusal disorder. Of the normal patients, 80% did not have joint or occlusal disorder and 20% had moderate disorder, showing that Rheumatoid Arthritis in dentulous patients, partially edentulous or those with partial or total prosthesis, have a greater joint involvement, resulting in joint interferences such as clicks and crepitation, more pronounced than in normal patients also edentulous or with prosthesis, going against the results attained by Ogus^20^, who did not find any significant correlation between dental malrelation with greater predisposition to having Temporomandibular Joint abnormalities. As to the assessment of the anamnesis, dysfunctional and occlusal components of the Helkimo's index, according to age range, a statistical correlation between more severe grades of anamnesis, dysfunctional and occlusal components and older age ranges, and less severe grades and younger age was not seen for anamnesis and dysfunction components, it was only seen for the occlusal component.

In analyzing the craniomandibular index and its components, we observed mean values that were statistically higher in the group of patients with Rheumatoid Arthritis, who had more severe joint involvement when compared to the values attained from the disease-free patients; thus corroborating Gil and Nakamae' study^19^, in which they proved the safety of using the craniomandibular index when assessing craniomandibular signs and symptoms. Since the literature is not very abundant in references as to the use of such index, the values hereby attained could not be compared to those from other studies; we were only able to compare the large value differences among patients with (0.12) and those without Rheumatoid Arthritis (0.02).

As to assessing the gender variant in relation to craniomandibular index values, we did not find statistically significant difference, and the same happened in regards to the age variable. Such data could not be correlated with literature data because of its scarcity.

Both indexes had statistically significant results as to the diagnosis of temporomandibular disorders when applied to patients with and those without Rheumatoid Arthritis. We noticed that TMJD prevalence was higher in the Rheumatoid Arthritis group (98.6% for the Helkimo's index and 50% for the craniomandibular index). Results found show a greater prevalence of temporomandibular disorder in patients with systemic Rheumatoid Arthritis, showing its influence on Temporomandibular Joint.

## CONCLUSIONS

1. The Helkimo's and Craniomandibular (CMI) indexes are able to detect temporomandibular disorders in patients with RA, nonetheless, the Helkimo's index is more accurate;
2. There was a significant difference between RA and normal patients as far as TMJD is concerned for both indices;
3. TMJD prevalence was higher in the group with RA when compared to patients without RA for both indices.
